# Prolonged moderate to vigorous physical activity may lead to a decline in cognitive performance: a Mendelian randomization study

**DOI:** 10.3389/fnagi.2024.1403464

**Published:** 2024-09-20

**Authors:** Yutao Li, Chenyi Fu, Honglin Song, Zhenhang Zhang, Tianbiao Liu

**Affiliations:** ^1^School of Physical Education and Sports Science, South China Normal University, Guangzhou, China; ^2^College of Physical Education and Sports, Beijing Normal University, Beijing, China

**Keywords:** moderate to vigorous physical activity, cognitive performance, Mendelian randomization, multivariate MR analysis, epigenetics

## Abstract

**Objective:**

This study investigates the causal relationship between moderate to vigorous physical activity and cognitive performance.

**Methods:**

Genetic loci strongly related to moderate to vigorous physical activity from genome-wide association studies were used as instrumental variables. These were combined with genetic data on cognitive performance from different Genome-Wide Association Study (GWAS) to conduct a two-sample Mendelian randomization analysis. The primary analysis used inverse variance weighting within a random effects model, supplemented by weighted median estimation, MR-Egger regression and other methods, with results expressed as Beta coefficient.

**Results:**

This study selected 19 SNPs closely related to physical activity as instrumental variables. The multiplicative random-effects Inverse-Variance Weighted (IVW) analysis revealed that moderate to vigorous physical activity was negatively associated with cognitive performance (Beta = −0.551; OR = 0.58; 95% CI: 0.46–0.72; *p* < 0.001). Consistent results were obtained using the fixed effects IVW model (Beta = −0.551; OR = 0.58; 95% CI: 0.52–0.63; *p* < 0.001), weighted median (Beta = −0.424; OR = 0.65; 95% CI: 0.55–0.78; *p* < 0.001), simple mode (Beta = −0.467; OR = 0.63; 95% CI: 0.44–0.90; *p* < 0.001), and weighted mode (Beta = −0.504; OR = 0.60; 95% CI: 0.44–0.83; *p* < 0.001). After adjusting for BMI, smoking, sleep duration, and alcohol intake frequency, the multivariate MR analysis also showed a significant association between genetically predicted MVPA and cognitive performance, with Beta of −0.599 and OR = 0.55 (95% CI: 0.44–0.69; *p* < 0.001).

**Conclusion:**

The findings of this study indicate that genetically predicted moderate to vigorous physical activity may be associated with a decline in cognitive performance.

## Introduction

A growing body of research suggests that physical activity has a significant impact on cognitive performance. However, establishing a causal relationship between physical activity and cognitive performance remains challenging due to confounding factors such as socioeconomic status, education, and access to healthcare. Cognitive performance refers to the brain’s ability to process and interpret information, encompassing essential functions such as reasoning, attention, thinking, reading, and learning (Gale et al., 2010). This critical aspect of brain function is linked to various health outcomes, including morbidity, mortality, mental disorders, coronary heart disease (CHD), and certain cancers (Gale et al., 2010; Hart et al., 2003; Jaycox et al., 2009; Lawlor et al., 2008a). In recent years, the prevalence of risk factors such as aging, obesity, and unhealthy lifestyles (e.g., smoking, excessive alcohol consumption, irregular sleep patterns, etc.) has been associated with a decline in cognitive performance (Albert et al., 2011; Colcombe and Kramer, 2003; Salthouse, 1996; Elias et al., 2003; Gunstad et al., 2007; Smith et al., 2011; Eckardt et al., 1998; Strine and Chapman, 2005). It is believed that individuals who engage in frequent physical activity in their early years are more likely to maintain a more active lifestyle in the middle and even old age. Additionally, research indicates that physical exercise and activity during early life can lead to beneficial changes in the brain, reducing the likelihood of cognitive deficits later in life (Sabia et al., 2017; Scarmeas and Stern, 2004). Therefore, identifying interventions or factors that can mitigate cognitive decline early is crucial for reducing the risk of cognitive-related diseases.

Physical activities encompass a range of daily activities such as walking, work exertion (da Silva et al., 2018), recreational activities, household chores, and more intense physical activities like running, dancing, and competitive sports. Work-related physical activity contributes most of overall physical activity, particularly for lower-income groups with household incomes below the median of $36,000 (Matsushita et al., 2015; Suminski et al., 2011). Numerous studies have consistently demonstrated the positive effects of moderate physical activity on cognitive performance (Cotman and Berchtold, 2002; Ngandu et al., 2015; Chen et al., 2016; Weaver and Jaeggi, 2021; Wollseiffen et al., 2016). For instance, a large cohort study from Brazil found that adolescents engaging in moderate to vigorous physical activity (MVPA) tend to exhibit higher cognitive performance (Esteban-Cornejo et al., 2015). Additionally, research indicates that older adults with higher levels of physical activity experience a slower decline in cognitive performance (Albert et al., 2011; Gauthier et al., 2011). However, a systematic review of studies found that the effects of physical activity on cognitive performance are inconsistent, with limited conclusive evidence supporting a positive impact of physical activity on cognition. It is inaccurate to generalize that physical activity universally improves cognitive performance, as the type, amount, frequency, and duration of physical activity can have varying effects (Barbosa et al., 2020; Donnelly et al., 2016; Latino and Tafuri, 2023). Some studies suggest that high-intensity physical activity may lead to a decline in cognitive performance (Samuel et al., 2017). Even the most rigorous studies on brain cognitive health, such as those employing population-based cohorts with longitudinal follow-up or natural experiments, may exhibit biases. These biases can arise from attrition (e.g., higher dropout rates among individuals with the outcome of interest), self-selection (e.g., lifestyle and exercise behavior changes following cognitive impairment in older adults), and residual confounding (Besser et al., 2021). In summary, the evidence from the aforementioned studies primarily relies on observational correlations, leaving the causal relationship between physical activity and cognitive performance uncertain.

While randomized controlled trials (RCTs) are the gold standard for establishing causal relationships, ethical and practical challenges often limit their feasibility in studying the effects of exercise on cognitive performance. Despite the established correlation between physical activity and cognitive performance, causality remains difficult to ascertain due to potential confounding factors. To overcome these limitations, this study employs Mendelian Randomization (MR) to investigate the causal relationship between physical activity and cognitive performance. MR leverages the principle of random gene allocation, as outlined by Mendel’s second law, to assess causal relationships between exposures and outcomes (Smith and Ebrahim, 2003). By using genetic variants associated with specific exposures as instrumental variables, MR minimizes confounding and reverse causality biases that often challenge traditional observational studies (Richmond and Smith, 2022).

In this study, a two-sample MR approach was utilized, drawing on data from Genome-Wide Association Studies (GWAS) to assess the independent role of moderate-to-vigorous physical activity (MVPA) in cognitive performance (Hu et al., 2023). Additionally, a multivariable Mendelian Randomization (MVMR) analysis was performed to evaluate the causal effect of MVPA on cognitive performance while adjusting for other risk factors, including BMI, smoking, alcohol consumption, and sleep patterns.

## Methods

### Study design

**Figure 1:**
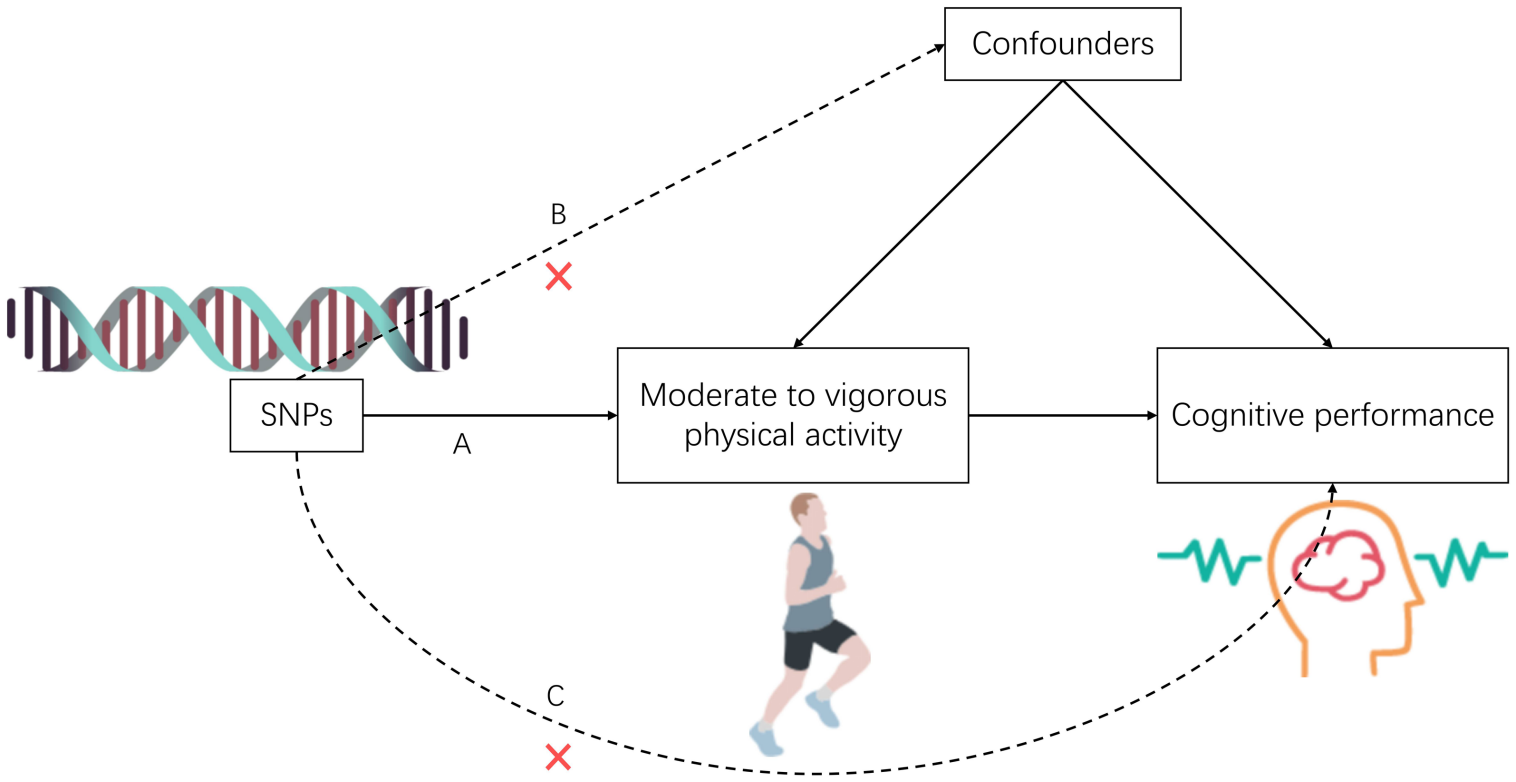
The overview of the study design.

The study comprised five components: (1) Identifying genetic variants as instrumental SNPs for exposure data through screening; (2) Collecting instrumental SNPs for outcome data from a genome-wide association study database focused on cognitive performance; (3) Integrating instrumental SNPs from the exposure and outcome datasets; (4) Conducting two-sample Mendelian randomization analyses and multivariate Mendelian randomization analyses; and (5) Conducting sensitivity analyses to evaluate the robustness of the Mendelian randomization analysis. To ensure valid causal estimates from the Mendelian analyses, the selected single nucleotide polymorphisms (SNPs) as genetic instruments for physical activity must satisfy three assumptions: (A) strong association with physical activity, (B) no association with confounding factors of the exposure-outcome relationship, and (C) exclusive influence on cognitive performance risk through the exposure of physical activity (see Figure 1).

### Data sources for physical activity and cognitive performance

Genetic instruments for MVPA were obtained from a GWAS conducted by Klimentidis et al. (2018) on a sample of 377,234 individuals in Europe descent, comprising both males and females. Physical activity levels were assessed using self-reported data collected via a touchscreen questionnaire. Participants were asked: “In a typical week, how many days did you do 10 min or more of moderate activities like carrying light loads or cycling at a normal pace (excluding walking)?” For vigorous activities, they were asked: “In a typical week, how many days did you do 10 min or more of vigorous activities like fast cycling, aerobics, or heavy lifting?” Those who reported engaging in such activities on at least 1 day were then asked how many minutes they usually spent on these daily activities, including work, leisure, travel, and housework (Klimentidis et al., 2018). MVPA was quantified by multiplying the total number of minutes of moderate physical activity per week by four and the total number of minutes of vigorous physical activity per week by eight, corresponding to their metabolic equivalents, as previously described (Bassett, 2003; Ekelund et al., 2006). Data are available for download on the ieu website at https://gwas.mrcieu.ac.uk/datasets/ebi-a-GCST006097/.

Summary-level genetic data for cognitive performance was collected by the Cognitive Genomics Consortium (COGENT) (Lee et al., 2018). The COGENT consortium performed a GWAS for cognitive performance in 257,841 individuals, and these results were meta-analyzed with published results from the UK Biobank (Lee et al., 2018). The participants included in the study were individuals of European descent, ranging in age from 16 to 102 years. Cognitive performance was measured using the first unrotated component derived from at least three neuropsychological tests. These tests include digit span for working memory, logical memory for verbal declarative memory, and digit symbol coding for processing speed. Population-specific genotypic principal components were included as covariates (Trampush et al., 2017). Fluid intelligence was assessed using the verbal numerical reasoning test in the UK Biobank, which evaluates participants’ ability in numerical reasoning and verbal comprehension. Estimates are reported in standard deviation (SD) units per SNP.

Additionally, the UK Biobank provides data on three other cognitive tests: reaction time, pairs matching, and prospective memory (Lee et al., 2018). The cohorts participating in the GWAS mentioned above obtained ethics approval from the respective ethical review boards and informed written consent from all participants (Zonneveld et al., 2023). Data on Cognitive Performance can be downloaded from https://gwas.mrcieu.ac.uk/datasets/ebi-a-GCST006572/.

### SNP selection

This study defined a genome-wide significance level of *P* < 5 × 10^−8^.To filter out weak instrumental variables, the potency of each SNP as an instrumental variable was assessed using the formula provided in a previous study to calculate the F statistic (Papadimitriou et al., 2020). An *F* > 10 was required for effective selection. SNPs with significant linkage disequilibrium needed to be removed to satisfy the independence assumption. An r^2^ value of 1 indicates complete linkage disequilibrium between the two SNPs, while an r^2^ value of 0 indicates complete linkage balance. In this study, the parameter r^2^ was set to 0.001, and kb was set to 10,000. This represents the removal of SNPs with an r^2^ value greater than 0.001 within a range of 10,000 kb. Consequently, instrumental variables with no linkage effect were screened from the different MVPA data.

### Statistical analysis

The principal analysis was conducted with an inverse-variance weighted (IVW) multiplicative random-effects model. A series of sensitivity analyses were performed to calculate the error variance using a fixed IVW, an MR-Egger regression, a weighted median, a simple mode, and a weighted mode to account for potential invalid instrument bias or pleiotropy (Hu et al., 2023). Invalid instrument bias was assessed using the weighted median model, which provided consistent results even when up to 50% of the weight was derived from invalid SNPs (Burgess et al., 2017). MR-Egger regression analysis was used to detect and correct for directional pleiotropy, and the intercept from MR-Egger was assessed to determine whether horizontal pleiotropy existed (Bowden et al., 2015). Funnel plots were also used to identify potential horizontal pleiotropy by assessing asymmetry. A multivariable MR analysis was conducted to adjust for BMI and smoking as adjustment factors. Heterogeneity among the included SNPs was assessed using Cochrane’s Q value. SNPs individually were examined for their impact on the overall causal estimate by performing a leave-one-out sensitivity analysis. The “TwoSampleMR” and “MVMR” packages in R version 4.2.3 were used for all analyses.

## Results

After confirming a strong correlation and resolving any potential chain imbalance, 1.5 million items associated with MVPA were extracted for further analysis. Table 1 presents the basic characteristics of the 19 SNPs linked to physical activity. In this table, “Chr” denotes chromosomal information, “NEA/EA” represents the non-effect and effect alleles, respectively, and “Beta” indicates the effect size of the MVPA-associated SNPs. The F statistics corresponding to individual SNPs in this study range from 30 to 52, with all *F* > 10. This suggests that the instrumental variables used are robust, thereby reducing the likelihood of weak instrument bias and reinforcing the reliability of the study’s findings.

**Table 1:** Characteristics of SNPs associated with physical activity.

SNP	chr	EA	NEA	Beta	SE	*P*	*F*
rs2942127	1	A	G	−0.016	0.003	3.30E-08	31
rs1974771	2	A	G	0.021	0.004	6.60E-09	34
rs2035562	3	G	A	0.014	0.002	3.90E-09	35
rs2114286	3	G	A	0.012	0.002	3.30E-08	31
rs877483	3	C	T	−0.012	0.002	4.00E-08	30
rs1972763	4	T	C	−0.013	0.002	3.30E-08	31
rs77742115	5	C	T	0.018	0.003	9.60E-09	33
rs2854277	6	T	C	−0.032	0.005	2.60E-10	40
rs1043595	7	A	G	−0.014	0.002	4.30E-09	34
rs1186721	7	A	G	0.013	0.002	4.40E-08	30
rs7804463	7	C	T	−0.015	0.002	1.20E-11	46
rs921915	7	C	T	0.014	0.002	5.70E-10	38
rs2988004	9	G	T	0.013	0.002	4.10E-09	35
rs7326482	13	T	G	0.013	0.002	1.60E-08	32
rs10145335	14	A	G	0.014	0.003	2.70E-08	31
rs12912808	15	T	C	−0.018	0.003	1.70E-08	32
rs4886868	15	G	T	0.012	0.002	3.50E-08	30
rs429358	19	C	T	0.022	0.003	6.10E-13	52
rs1921981	21	A	G	−0.013	0.002	3.80E-08	30

Chr, chromosome; EA, effect allele; NEA, non-effect allele; SE, standard error; SNP, single nucleotide polymorphisms.

**Figure 2:**
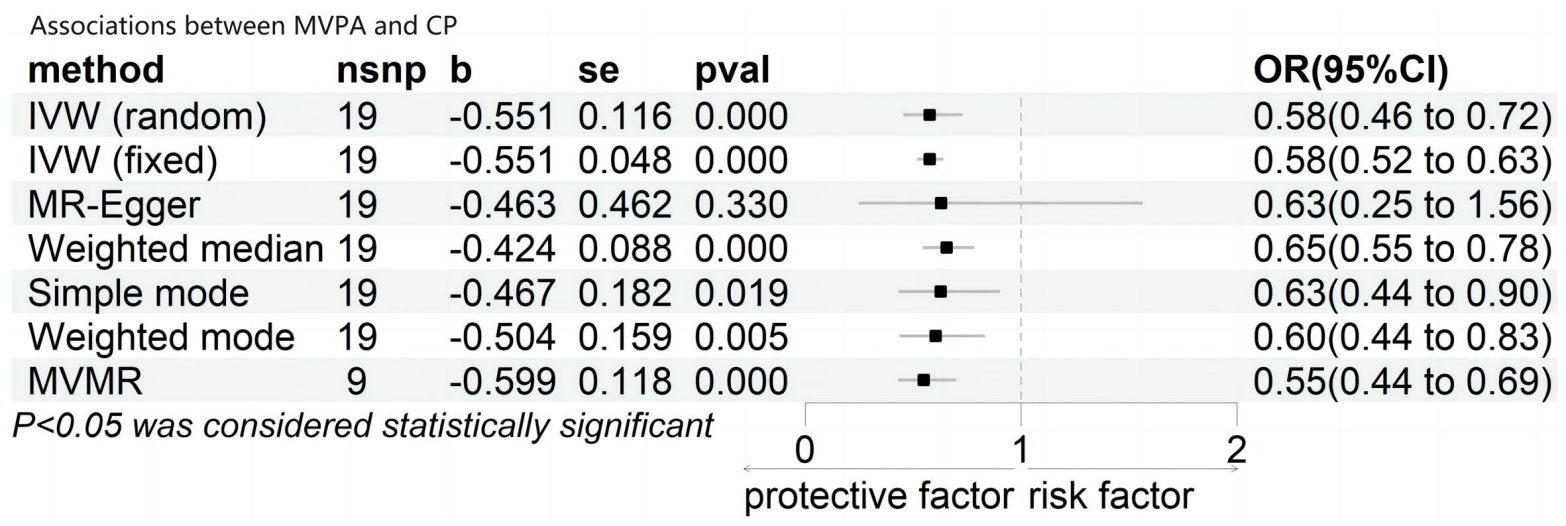
Associations between MVPA and cognitive performance. CI, confidence interval; OR, odd ratio; b, Beta; nsnp, number of SNP nsnp number.

As shown in Figure 2, genetically predicted MVPA had a significant effect on cognitive performance under the multiplicative random-effects inverse-variance weighted (IVW) model, with a Beta coefficient of −0.551 and odds ratio (OR) of 0.58 (95% confidence interval [CI]: 0.46–0.72; *p* = 0.000). Consistent results were obtained using the fixed effects IVW model (OR = 0.58; 95% CI: 0.52–0.63; *p* < 0.001), weighted median (Beta = −0.424; OR = 0.65; 95% CI: 0.55–0.78; *p* < 0.001), simple mode (Beta = −0.467; OR = 0.63; 95% CI: 0.44–0.90; *p* < 0.001), and weighted mode (Beta = −0.0.504; OR = 0.60; 95% CI: 0.44–0.83; *p* < 0.001). After adjusting for BMI, smoking, sleep duration, and alcohol intake frequency, the multivariate MR analysis also showed a significant association between genetically predicted MVPA and cognitive performance, with a Beta coefficient of −0.599 and OR = 0.55 (95% CI: 0.44–0.69; *p* < 0.001). However, only the MR-Egger model results indicated that MVPA did not significantly affect cognitive performance, with a Beta coefficient of −0.0.463 and OR of 0.63 (95% CI: 0.25–1.56; *p* = 0.330).

**Figure 3:**
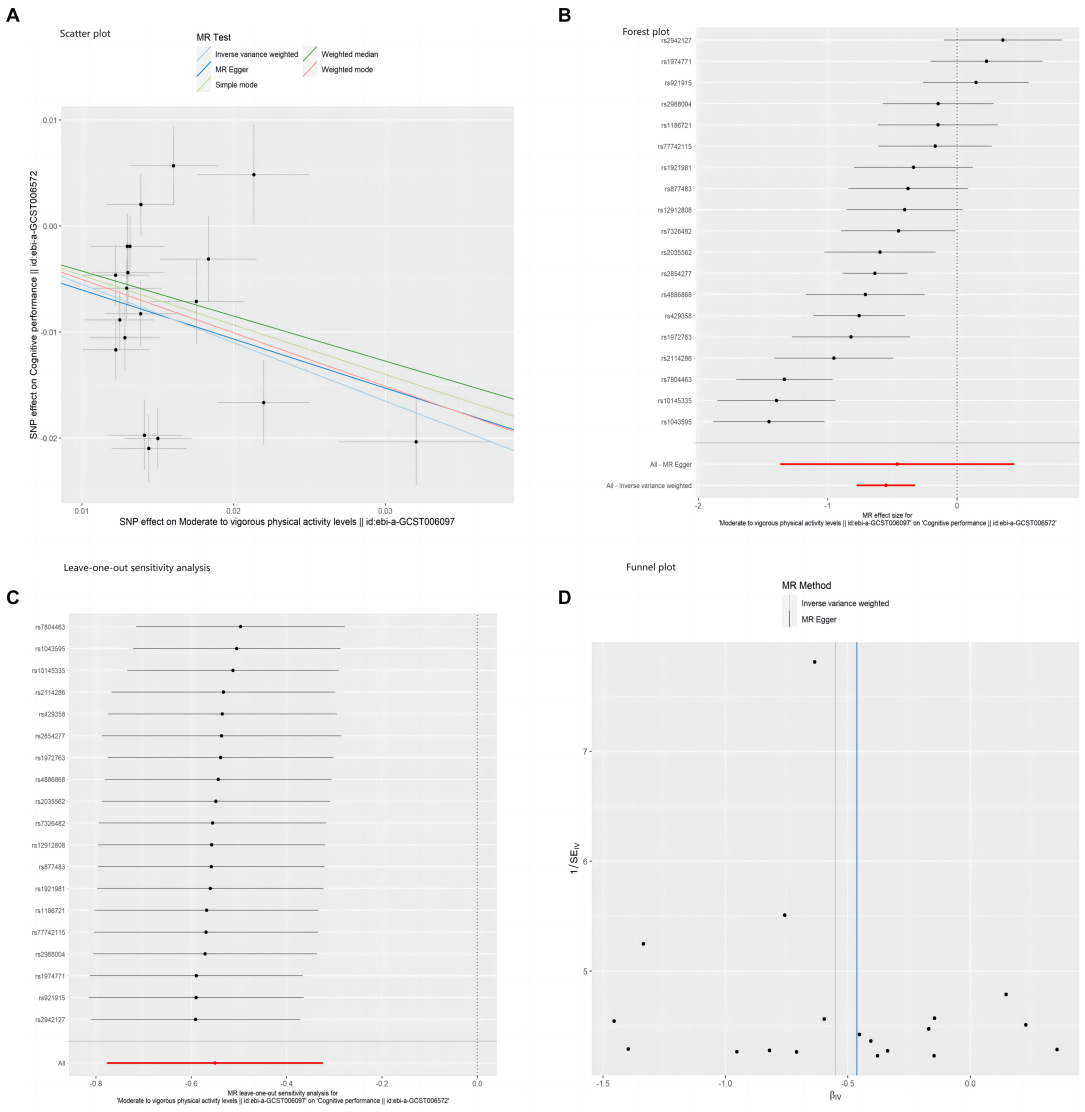
Scatter plot **(A)**, forest plot **(B)**, leave-one-out sensitivity analysis **(C)**, and funnel plot **(D)** of the association of MVPA with cognitive performance.

In addition, the sensitivity analysis results indicated the absence of pleiotropy in this study (*p* = 0.85). The MR-PRESSO test (*p* = 0.193) also yielded consistent results, as visualized in Figure 3D, where the funnel plot for the IVW method exhibited symmetry, indicating no presence of horizontal pleiotropy. Furthermore, scatter plots and forest plots based on all single nucleotide polymorphisms (SNPs) are presented in Figures 3A,B, respectively. In contrast, Figure 3C displays the sensitivity analysis results for the leave-one-out method.

## Discussion

This study employed a two-sample MR analysis using genetic instruments derived from large-scale genome-wide association studies data to strengthen the causal inference. MR leverages gene variation associated with a target exposure to estimate causality. By relying on the random assignment of genetic variation from parents and offspring, MR helps overcome the limitations of observational studies, thereby reducing the potential for confounding (Lawlor et al., 2008b). Since genetic variation is determined at conception, the method minimizes the risk of reverse causation. If MVPA has a causal effect on cognitive performance, genetic variants associated with physical activity should also be linked to cognitive performance. The study investigates the relationship between MVPA and cognitive performance. The approach utilizes genetic information to evaluate the causal relationship between MVPA and cognitive performance, mitigating confounding factors. The findings suggest that MVPA may have a detrimental effect on cognitive performance. Despite being widely recognized for its benefits in improving cognitive performance and preventing cognitive impairment, this study indicates that there might not be a direct causal relationship between physical activity and cognitive performance enhancement. Therefore, recommendations to promote physical activity may have an uncertain impact on improving cognitive performance improvement.

The mechanisms by which physical activity enhances cognitive performance can be explained on macro and cellular levels. On a macro level, individuals who engage in regular physical activity are often involved in cognitively stimulating activities such as gardening, hiking, or outdoor sports. These activities frequently include cognitive components, such as reading about outdoor sports, and increase social interaction, further stimulating cognitive processes and enhancing cognitive performance (Robitaille et al., 2014; Berchtold et al., 2005; Hall et al., 2000). Cross-sectional studies have consistently shown a positive correlation between physical activity and cognitive performance (Reas et al., 2019). Aerobic exercise and strength training have improved executive function, memory, and verbal fluency. Longitudinal studies also suggest that more excellent physical activity is associated with a slower decline in overall cognitive performance, memory, attention, and processing speed (Wang et al., 2023). On a cellular and physiological level, physical activity may exert its effects through several mechanisms. It stimulates the production of brain-derived neurotrophic factor (BDNF), crucial for cognitive performance and brain development (Khan and Hillman, 2014). BDNF supports neuronal cell survival, facilitates synaptic plasticity, and promotes neurogenesis and neuronal differentiation, all contributing to improved cognitive performance (Griffin et al., 2011). Physical activity leads to a rapid and sustained increase in mature BDNF protein and signaling in the brain (Hall et al., 2000; Berchtold et al., 2005), which in turn raises serum concentrations of these neurotrophins and enhances cognitive performance through regulatory mechanisms (Tyrrell and Pereira, 1980). In a study examining the effects of different interventions on BDNF levels in healthy older adults, 19 participants engaged in 35 min of physical exercise, cognitive training, and mindfulness exercises. The study compared the changes in serum BDNF levels among the three intervention groups. The results revealed a significantly higher increase in serum BDNF levels in the physical exercise group compared to the cognitive training and positive thinking group (Hakansson et al., 2017).

Research consistently highlights the positive impact of physical activity on the hippocampus, a brain region crucial for declarative memory consolidation, spatial orientation, and emotion regulation. Biologically, there is a positive correlation between hippocampal size and cognitive abilities, with a larger hippocampal volume generally being advantageous (Anblagan et al., 2018; Konishi et al., 2017; Reuben et al., 2011). Studies have shown that physical activity can increase hippocampal size in adults, thereby enhancing cognitive performance (Liu and Nusslock, 2018). The hippocampus, located deep within the brain’s medial temporal lobe, plays a critical role in cognitive performance (El-Falougy and Benuska, 2006; Geinisman et al., 1995). Impairment in the structure and function of the hippocampus can contribute to cognitive performance declines and cognitive deficit development (Geinisman et al., 1995). In a randomized controlled trial involving 120 older adults, aerobic exercise training increased the volume of the anterior hippocampus, leading to improvements in cognitive performance. The study observed a 2% increase in hippocampal volume due to exercise training, counteracting age-related volume loss and its associated adverse effects (Erickson et al., 2011). A study focusing on physically inactive older adults found that a physical activity intervention, including increased daily walking, increased hippocampal volume (Varma et al., 2015). Regarding the relationship between the BDNF gene and the hippocampus, the BDNF gene contains two main alleles that produce varying amounts of protein products. These products regulate neurons in the brain, including those in the hippocampus, influencing hippocampal volume and cognitive behavioral traits (Frodl et al., 2014; Marrocco et al., 2020; Palasz et al., 2020; Vilor-Tejedor et al., 2020).

However, there is ongoing debate regarding the effects of physical activity on cognitive performance. Research has shown that the relationship between the intensity, type, and amount of exercise and its impact on BDNF levels or cognitive performance is not always straightforward. While several physiological mechanisms suggest that physical activity may enhance cognition, others propose that it could negatively affect cognitive performance. Given that these mechanisms are complex and sometimes conflicting, current scientific understanding and technology have yet to describe the relationship between physical activity and cognition precisely.

Certain studies have indicated that intense and strenuous exercise can disrupt the body’s metabolism and physiological processes, negatively impacting cognitive performance (Aguiló et al., 2005). During physical activity, the rapid metabolism of oxygen produces reactive oxygen species (ROS) as a metabolic by-product. High levels of ROS accumulated during continuous intense exercise can lead to oxidative damage and increase neuronal mortality (Radak et al., 2016). While moderate physical activity enhances the body’s antioxidant defense system, excessive ROS generated from intense exercise may detrimentally affect cognitive performance if accumulated in excess (Mastaloudis et al., 2001). A cohort study on adolescents found that those engaged in high-intensity physical activity had lower cognitive scores, suggesting a negative impact of such exercise on cognitive performance during adolescence (Esteban-Cornejo et al., 2015). Additionally, a population-based study reported that extending exercise beyond 1 h could decrease cognitive performance. This effect might be linked to dehydration or hypoglycemia during acute exercise, as increased sweating and elevated body temperature can lead to water and electrolyte loss, impairing cognitive performance such as decision-making and perceptual tasks (Brisswalter et al., 2002). In a study involving older adults, 105 participants were randomly assigned to high-intensity interval training (*n* = 33), moderate-intensity continuous training (*n* = 24), or a control group (*n* = 48). The findings suggested that high-intensity interval exercise might negatively affect the hippocampus and overall cognitive performance (Pani et al., 2021). Furthermore, a large, nationally representative six-year longitudinal study on school sports participation revealed that increased participation had a negative effect on students’ performance in standardized tests (Marsh and Kleitman, 2003). Another study assessing an intensive physical education program found that intense intermittent aerobic exercise negatively impacted children’s numerical speed and accuracy in cognitive tasks such as simple addition problems (Travlos, 2010).

Given the inconsistent evidence regarding the causal relationship between physical activity and cognitive performance in previous studies, the present study conducted an MR analysis to explore this relationship at the genetic level. Additionally, external factors such as age, environment, lifestyle, diet, and comorbidities interact with the genome through epigenetic modifications. These modifications regulate gene expression across various tissues without altering the underlying DNA sequence (Barati et al., 2022). Research has shown that changes in the epigenome can contribute to cognitive disorders (Strafella et al., 2018). Thus, epigenetic changes might influence the relationship between physical activity-related SNPs and cognitive performance. To account for potential epigenetic influences, relevant risk factors such as sleep duration, alcohol consumption, BMI, and smoking were identified through a literature review and adjusted for in a multivariate MR analysis. The findings of this study indicate that genetically predicted MVPA may be associated with a decline in cognitive performance.

However, several potential positive biological adaptation pathways suggest that physical activity may help maintain cognitive performance throughout individual development. Aerobic exercise can modulate neurotransmission, promote angiogenesis, enhance neurotrophic factors and synaptic plasticity, and facilitate neurogenesis, thereby improving cerebrovascular function (Blomstrand et al., 1989; Isaacs et al., 1992; Farmer et al., 2004). The health benefits of physical activity are well-documented, including improved physical fitness, reduced disease risk, and enhanced quality of life. In both adults and adolescents, increased physical activity is strongly associated with positive subjective health outcomes (Mountjoy et al., 2011). Self-reported health status, which encompasses illness experience, physical ability, health behaviors, living situation, and self-esteem, has been shown to predict mortality (Idler and Benyamini, 1997). Regular physical activity can lower mortality risk in coronary heart disease patients and improve their functional and emotional wellbeing (Rodriguez et al., 1994; Haapanen et al., 1997; Lee et al., 2000). Studies on individuals with diabetes have shown moderate-intensity physical activity enhances metabolism (Lee et al., 2015), while high-intensity physical activity is particularly effective for cardiovascular health and blood sugar control (Jelleyman et al., 2015).

Furthermore, increasing physical activity is an effective strategy for improving the physical and mental health of the elderly (Scarmeas et al., 2001; Gilovich et al., 2002; Feldman et al., 2015). Physical activity benefits the elderly by enhancing physical and mental health, social adaptation, activity levels, and nutritional status. It can extend healthy life expectancy, improve quality of life, and boost overall wellbeing (Cui et al., 2021). For example, a meta-analysis of 36 studies found that elderly individuals participating in physical activity programs reported significantly higher levels of mental health, including emotional wellbeing, self-perception, and life satisfaction, compared to non-participants (Netz et al., 2005). Given the overall health benefits of physical activity, promoting it in the general population remains justified despite concerns about reduced cognitive performance. However, caution is advised with prolonged MVPA to mitigate potential negative impacts on cognitive performance. Future research should explore the effects of different types and intensities of exercise at the genetic level to identify the most beneficial forms for cognitive performance and overall health while minimizing the risk of cognitive decline.

It is important to acknowledge certain limitations of this study. Firstly, the research focused exclusively on the unidirectional impact of physical activity on cognitive performance. However, literature suggests a potentially more complex bidirectional relationship between physical activity and cognitive performance. For instance, some studies have found that a decline in cognitive resources is associated with lower levels of moderate physical activity (Cheval et al., 2020) and that a bidirectional relationship exists between physical activity and executive function in older adults (Daly et al., 2015). Moreover, cognitive resources have been shown to moderate the negative impact of perceived poor neighborhood conditions on older adults’ self-reported physical activity (Cheval et al., 2019). Future research should employ bidirectional Mendelian randomization to address these complexities to investigate the reciprocal causal relationship between physical activity and cognitive ability. This approach will provide a more nuanced understanding of how physical activity and cognitive performance interact and offer valuable scientific evidence for developing more effective public health policies and interventions.

## Data Availability

The original contributions presented in the study are included in the article/supplementary material, further inquiries can be directed to the corresponding author.
